# The Impact of Intestinal Inflammation on Nematode’s Excretory–Secretory Proteome

**DOI:** 10.3390/ijms241814127

**Published:** 2023-09-15

**Authors:** Marta Maruszewska-Cheruiyot, Ludmiła Szewczak, Katarzyna Krawczak-Wójcik, Magdalena Kierasińska, Michael Stear, Katarzyna Donskow-Łysoniewska

**Affiliations:** 1 Department of Experimental Immunotherapy, Faculty of Medicine, Lazarski University, Świeradowska 43, 02-662 Warsaw, Poland; m.maruszewska@lazarski.edu.pl; 2 Department of Parasitology, Institute of Functional Biology and Ecology, Faculty of Biology, University of Warsaw, Miecznikowa 1, 00-096 Warsaw, Poland; ludmila.szewczak@uw.edu.pl; 3 Department of Biomedical Sciences, Faculty of Physical Education, Józef Piłsudski University of Physical Education in Warsaw, Marymoncka 34, 00-968 Warsaw, Poland; katarzyna.krawczak@awf.edu.pl; 4 Department of Histology and Embryology, Medical University of Warsaw, Chałubinskiego 5, 02-004 Warsaw, Poland; glaczynska.magdalena@gmail.com; 5 Department of Animal, Plant and Soil Sciences, AgriBio, La Trobe University, Melbourne, VIC 3086, Australia; m.stear@latrobe.edu.au

**Keywords:** excretory–secretory proteins, nematode, parasite, colitis, inflammatory bowel disease, adaptation

## Abstract

Parasitic nematodes and their products are promising candidates for therapeutics against inflammatory bowel diseases (IBD). Two species of nematodes, the hookworm *Necator americanus* and the whipworm *Trichuis suis*, are being used in clinical treatment trials of IBD referred to as “helminth therapy”. *Heligmosomoides polygyrus* is a well-known model for human hookworm infections. Excretory–secretory (ES) products of *H. polygyrus* L4 stage that developed during colitis show a different immunomodulatory effect compared to the ES of *H. polgyrus* from healthy mice. The aim of the study was to evaluate excretory–secretory proteins produced by *H. polygyrus* L4 stage males and females that developed in the colitic milieu. Mass spectrometry was used to identify proteins. Blast2GO was used to investigate the functions of the discovered proteins. A total of 387 proteins were identified in the ES of *H. polygyrus* L4 males (HpC males), and 330 proteins were identified in the ES of L4 females that developed in the colitic milieu (HpC females). In contrast, only 200 proteins were identified in the ES of L4 males (Hp males) and 218 in the ES of L4 females (Hp females) that developed in control conditions. Most of the proteins (123) were detected in all groups. Unique proteins identified in the ES of HpC females included annexin, lysozyme-2, apyrase, and galectin. Venom allergen/Ancylostoma-secreted protein-like, transthyretin-like family proteins, and galectins were found in the secretome of HpC males but not in the secretome of control males. These molecules may be responsible for the therapeutic effects of nematodes in DSS-induced colitis.

## 1. Introduction

Parasites are mostly considered as pathogens that impact human and animal medicine and the economy all over the world. However, internal multicellular parasites are valuable suppliers of molecules with potentially useful applications in the treatment of several human diseases. To enhance our understanding of the proteins produced by helminths, proteomic analyses of excretory–secretory (ES) antigens are conducted. Based on the strong immunomodulatory abilities of helminths, two parasitic nematode species, *Necator americanus* and *Trichuis suis*, are used in experimental helminth therapy. To date, numerous clinical trials have been conducted using these parasites in the treatment of inflammatory bowel diseases (IBD) [1,2]. IBD, which includes Crohn’s disease and ulcerative colitis, is a chronic, idiopathic, relapsing inflammation of the gastrointestinal system, with a prevalence of 0.3% in western countries [3]. Experimental infection with nematodes seems to be well-tolerated in IBD patients. However, helminths are multicellular, relatively large organisms that produce a wide range of products, such as metabolic products, immunogenic proteins, and excretory–secretory molecules, including various immunomodulators. Until now, not much has been known about the precise molecules produced by nematodes during helminth therapy and the type of molecules to which patients are exposed. Detailed analyses of excretory–secretory products of nematodes used in helminth therapy have been already conducted [4,5]. However, nematodes investigated in these studies were developed in very controlled conditions, which do not always reflect the parasite milieu, especially during helminth therapy. The patient’s digestive tract in IBD is most often severely affected by the disease. Strong inflammation leads to significant changes in tissue structure as well as various factors such as cytokines, growth factors, and others. These molecules could influence parasite growth, viability, and fecundity as well as the molecules secreted by parasites.

An example of a well-described model organism for human hookworm infections is the murine intestinal parasite, *Heligmosomoides polygyrus*. Molecules produced by *H. polygyrus* strongly affect the host organism. A positive effect of infection with *H. polygyrus* has been demonstrated in mouse models of inflammatory bowel diseases, multiple sclerosis, and allergies, reflecting helminth therapy in humans [6,7,8]. Murine infections have helped to elucidate some mechanisms involved in helminth therapy. Furthermore, the effects of *H. polygyrus*-derived molecules are not limited to mice but also affect human cells [9]. Therefore, *H. polygyrus* may similarly influence an immune response in humans.

The composition of proteins produced by helminths depends on the conditions in which they live. This results from their adaptation to a new microenvironment. *H. polygyrus* is used in research on the mechanisms responsible for the therapeutic effect of deliberate infection in patients with inflammatory bowel diseases in clinical studies. *H. polygyrus* suppresses symptoms of colitis in the murine model. However, this therapeutic effect is accompanied by increased growth, survival, and reproduction of the parasite, as well as a much greater male-to-female ratio, all of which indicate adaptation of the parasite. This phenomenon was observed in a colitis model induced with dextran sulphate sodium (DSS) and is independent of the genetic background of the host [10]. Based on 2D proteomic analysis of the somatic antigens of L4-stage *H. polygyrus* that developed in colitic conditions, we observed strong differences in protein composition [11], and these changes were dependent of the sex of the nematode [12]. The immunomodulatory potential of nematodes that developed during colitis can be extremely different. Dendritic JAWSII cells cultured in the presence of L4 stage *H. polygyrus* from healthy mice show a therapeutic effect. In contrast, cells cultured with nematodes that developed during inflammation of the colon significantly worsened the condition of mice with DSS-induced colitis [13]. Our previous study of excretory–secretory products also indicated significant differences in ES composition between the male and female L4 stages of *H. polygyrus* [14]. Here, we evaluate the excretory–secretory proteins produced by both male and female nematodes of the L4 stage that developed in the colitic milieu using mass spectrometry and bioinformatic approaches.

## 2. Results

### 2.1. Variations in Excretory–Secretory Components between Female and Male H. polygyrus L4 Stage from Colitic and Control Environments

The analysis was carried out on protein sets generated from Mascot identification results. The analysis allowed us to identify 387 proteins in the ES of *H. polygyrus* L4 males (HpC males) and 330 proteins in the ES of L4 females that developed in a colitic milieu (HpC female). In comparison, only 200 proteins in the ES of L4 males (Hp males) and 218 in the ES of L4 females (Hp females) that developed in control conditions have been identified (Table S1). Many of the identified proteins (123) were detected in all groups.

**Figure 1:**
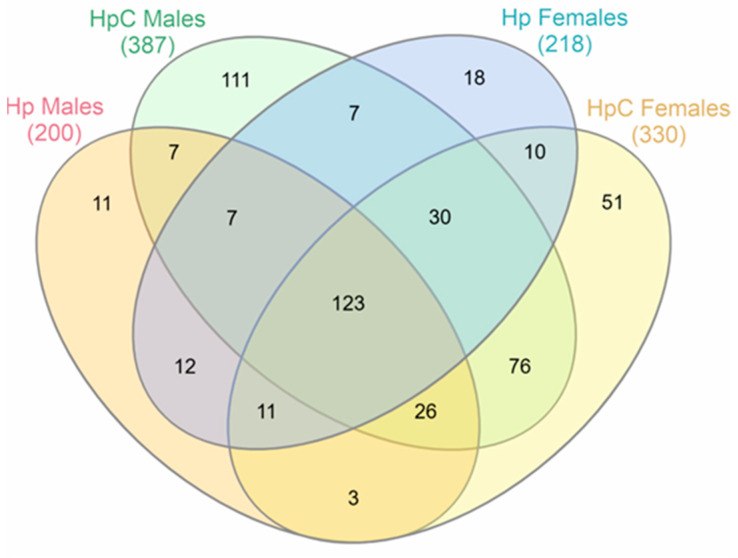
A Venn diagram presenting the distribution of molecules identified in the excretory–secretory antigen of *H. polygyrus* L4 stage females that developed in colitic conditions (HpC females), males that developed in colitic conditions (HpC males), females growing in control mice (Hp females), and males growing in control mice (Hp males). BALB/c male mice were given 3% dextran sulphate sodium (DSS) in drinking water and infected orally with 300 L3 *H. polygyrus*. Then, L4 stage nematodes were collected and ES generated.

Samples obtained from the HpC male and HpC female groups shared 255 proteins, which accounted for 65.8% of the proteins identified in HpC male-derived ES and 77.3% of proteins identified in HpC female-derived ES, respectively. Similarly, ES produced by the control Hp male and Hp female groups shared 153 proteins. This accounted for 76.5% of the proteins identified in Hp male-derived ES and 70.2% of the proteins in Hp male-derived ES. When ES from Hp L4 with the same sex but developed in different conditions were compared, HpC male and Hp male-derived ES shared 163 proteins. This represented 42.1% of the proteins found in HpC male-derived ES and 81.5% of the proteins identified in Hp male-derived ES. Furthermore, 174 proteins were shared by HpC female and Hp female-derived ES. This represented 44.5% of the proteins found in HpC female-derived ES and 80% of the proteins identified in Hp female-derived ES (Figure 1). In male and female ES obtained from *H. polygyrus* that developed in colitic conditions, 252 (77.8%) and 208 (76.25%) proteins had GO annotations, respectively. In the ES produced by nematodes that developed in control conditions, 117 (73.4%) male proteins and 114 (70.3%) female proteins were annotated.

### 2.2. GO Annotation of ES Proteins Produced by H. polygyrus L4 Stage That Developed in Colitic and Control Conditions

The highest number of terms in Gene Ontology (GO) annotations of excretory–secretory products in biological process category at level 2 was assigned in the HpC female group, with a total of 14 terms. Proteins from ES obtained from control females were assigned to 13 terms, while males that developed in both evaluated microenvironments were assigned to 12 terms.

**Figure 2:**
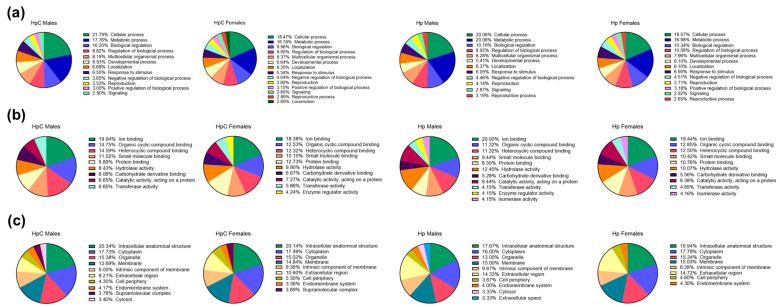
A comparison of Gene Ontology (GO) annotations for excretory–secretory proteins of male or female *H. polygyrus* L4 stage that developed during colitis or control conditions. BALB/c male mice were given 3% dextran sulphate sodium (DSS) in drinking water and infected orally with 300 L3 *H. polygyrus*. Then, L4 stage nematodes were collected and ES generated. Identified proteins were analyzed with the OmicsBox software version 2.4.4 based on the assigned biological process, level 2 (**a**); molecular function, level 3 (**b**); and cellular component, level 3 (**c**). *H. polygyrus* L4 stage males (HpC males) and females (HpC females) that developed during colitis, and males (Hp males) and females (Hp females) from control infection.

The most abundant terms in all examined samples were “cellular process” (GO:0009987), “metabolic process” (GO:0008152), “biological regulation” (GO:0065007), and “regulation of biological process” (GO:0050789). Despite significant differences in the number of proteins among examined groups, the variations in the percentage of proteins assigned to GO terms between samples were not considered crucial. However, molecules assigned to the term “locomotion” (GO:0040011) were only observed in the ES products of the HpC female group. None of the proteins from Hp male ES were matched to the term “positive regulation of biological process” (GO:0048518), while none of the proteins from HpC male group were assigned to the term “reproductive process” (GO:0022414) (Figure 2a).

Molecular function and cellular component categories at level 2 did not differ between evaluated samples. Therefore, we analyzed variations at level 3. Hp male proteins were assigned to 11 terms of molecular function, which was the highest number of terms among the four analyzed groups. Male HpC ES proteins were assigned to 9 terms, while females that developed in both tested microenvironments were assigned to 10 terms. Three of the most abundant terms related to molecular function at level 3 were “ion binding” (GO:0043167), “organic cyclic compound binding” (GO:0097159), and “heterocyclic compound binding” (GO:1901363). Almost half of the annotated proteins in ES HpC males were assigned to these three terms. Only molecules produced by HpC females and Hp males were assigned to “enzyme regulator activity” (GO:0030234). Molecules with “isomerase activity” (GO:0016853) were observed in the ES of nematodes under control conditions in both males and females (Figure 2b).

Annotated proteins produced by male nematodes, from both colitic and control conditions, were assigned to 10 terms of cellular components. In HpC females and Hp females, there were nine and eight terms, respectively. The most abundant terms related to the cellular components category were “intracellular anatomical structure” (GO:0005622), “cytoplasm” (GO:0005737), and “organelle” (GO:0043226). Molecules assigned to the “supramolecular complex” (GO:0099080) term have been observed in ES produced by nematodes that developed in colitic conditions, in both males and females. Proteins assigned to the “cytosol” term were present in the ES of male nematodes from colitic and control milieus. Only molecules produced by HpC females and Hp males were assigned to “enzyme regulator activity” GO:0030234. Additionally, molecules with “isomerase activity” (GO:0016853) have been observed in the ES of nematodes under control conditions in both males and females (Figure 2c).

### 2.3. Differences in Proteins’ Abundance between the ES of Nematodes That Developed in Colitic and Control Conditions

**Figure 3:**
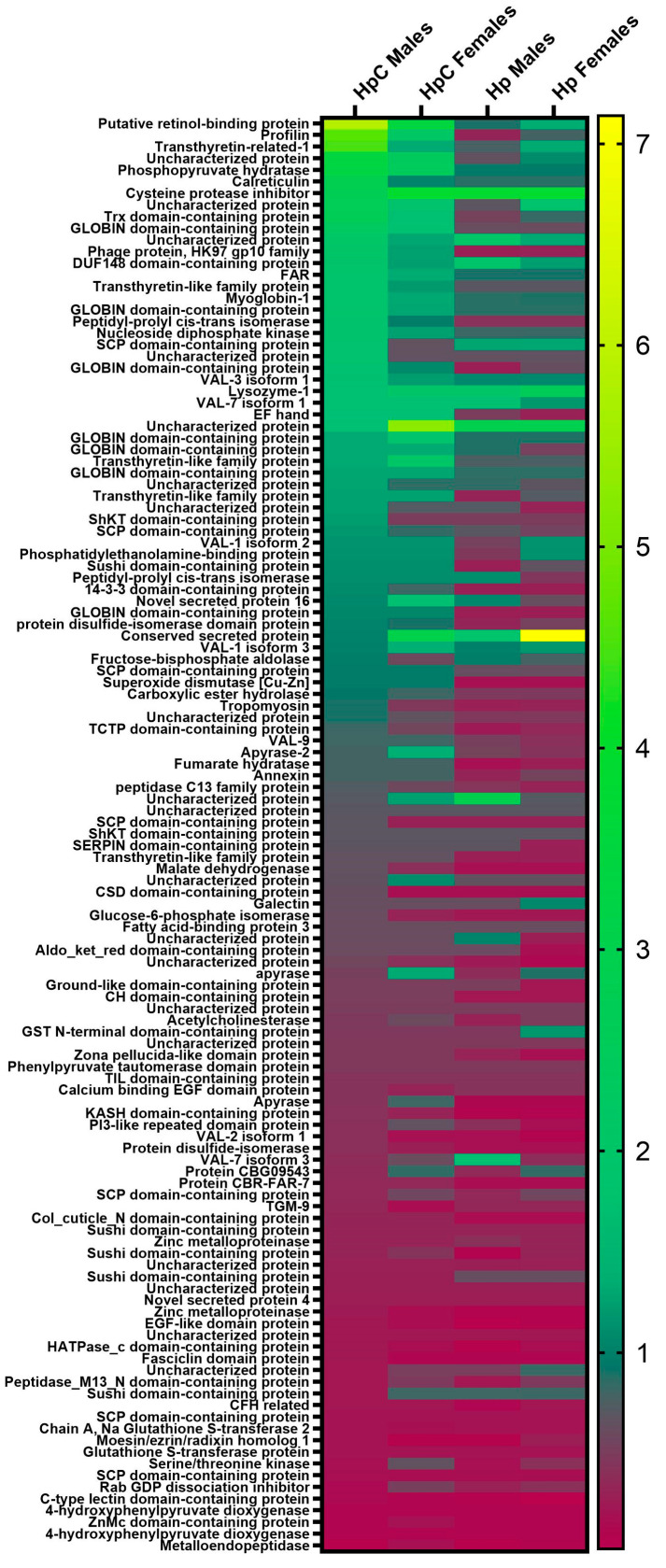
A comparison of exponentially modified protein abundance index (emPAI) of excretory–secretory molecules of *H. polygyrus* L4 stage that developed during colitis or control conditions. BALB/c male mice were given 3% dextran sulphate sodium (DSS) in drinking water and infected orally with 300 L3 *H. polygyrus*. Then, L4 stage nematodes were collected and ES generated. VAL—venom allergen/Ancylostoma-secreted protein-like, TGM—transforming growth factor (TGF)-β mimic, Trx—thioredoxin, FAR—fatty acid- and retinol-binding proteins, CH—calponin-homology, PI3—pepsin inhibitor-3. *H. polygyrus* L4 stage males (HpC males) and females (HpC females) developed during colitis, and males (Hp males) and females (Hp females) developed from control infection.

The abundance of proteins shared between all four studied groups varied with nematode development conditions and sex. The most abundant shared protein in HpC male ES was putative retinol-binding protein. This protein was also highly abundant in HpC female group with a lower exponentially modified protein abundance index (emPAI) in the ES of nematodes that developed in control conditions. In the shared protein set of HpC females, uncharacterized proteins predominated. Cysteine protease inhibitor and conserved secreted protein were the most abundant proteins shared between all groups, identified in the ES of Hp males and Hp females, respectively (Figure 3).

The most abundant unique proteins identified in the ES of male *H. polygyrus* L4 stage that developed in the colitic milieu and not detected in other evaluated groups were tubulin beta chain, EF-hand domain-containing protein, Hit domain-containing protein, cystatin domain-containing protein, and EF hand (Table 1). In the ES of females that developed in inflammatory conditions, the most abundant unique proteins were Aldo_ket_red domain-containing protein, SCP domain-containing protein, annexin, and A2M_N_2 domain-containing protein (endopeptidase inhibitor). The ES of HpC females contained various proteins with intestine microenvironment modulatory potential that have not been observed in ES sets obtained from other studied groups of nematodes. These proteins included lysozyme-2, apyrase, and galectin. Additionally (in HpC female ES), proteins with a lower estimated amount but also with possible immunomodulatory activity were also identified: calcium binding EGF domain protein, complement factor H-like, and EGF-like domain protein (Table 2). In HpC male ES, unique galectin was also identified (Table S2). All the unique proteins identified in the ES of HpC males and HpC females are listed in Table S1.

### 2.4. COG Categories of Distribution in the ES of H. polygyrus L4

In the EggNOG Clusters of Orthologous Genes (COG) annotation results, the most abundant term in the metabolism category was “carbohydrate transport and metabolism”. The same percentage of proteins was assigned to the “energy production and conversion” term in the HpC female group. Proteins assigned to the term “secondary metabolites biosynthesis, transport and catabolism” were present in colitic groups only.

The most abundant terms in the category “Information storage and processing” were translation, ribosomal structure, and biogenesis. The highest percentage of proteins from males that developed in both environments, compared to female groups, was annotated to this category. Proteins classified under the terms “RNA processing and modification” and “Chromatin structure and dynamics” were presented in the ES of nematodes that developed in the colitis milieu only. Proteins assigned to the subcategory “Nuclear structure” were present in the ES of HpC females only.

**Figure 4:**
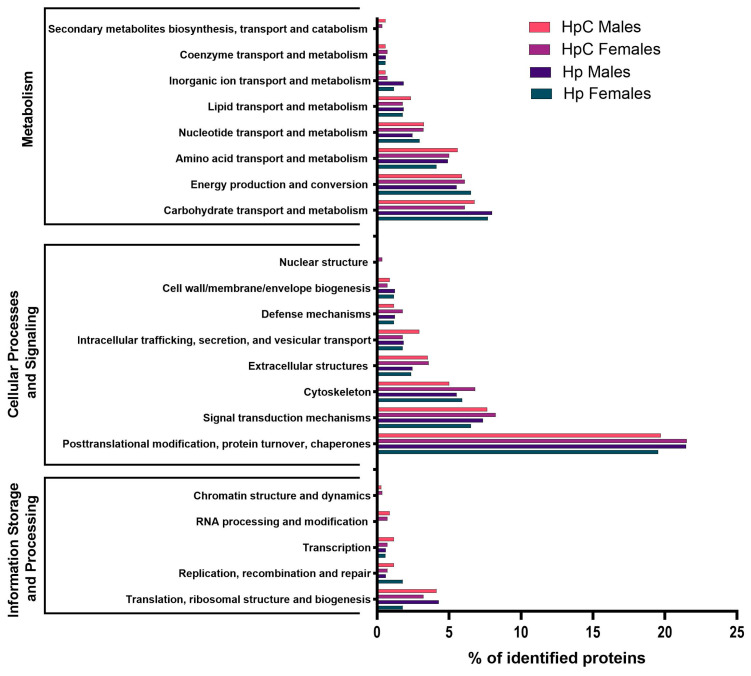
COG category distribution for ES proteins of *H. polygyrus* L4 stage males (HpC males), females (HpC females) that developed during colitis and males (Hp males) and females (Hp females) from control infection. BALB/c male mice were administered 3% dextran sulphate sodium (DSS) in drinking water and infected orally with 300 L3 *H. polygyrus*. Then, L4 stage nematodes were collected and ES generated.

Most of the proteins classified as “Cellular process and signaling” were assigned to the term “Translation, ribosomal structure and biogenesis”, with a higher percentage of proteins identified in the ES products of male nematode groups than in those of females from both evaluated milieus. Proteins assigned to the subcategories “Chromatin structure and dynamics” and “RNA processing and modification” were presented in the ES of nematodes that developed in an inflammatory microenvironment only (Figure 4). In the ES of *H. polygyrus* L4 stage males and females that developed during colitis, they constituted 22% and 24% of the proteins, respectively, and were classified as proteins of unknown function. In control groups, 27% of identified proteins in males and 30% in females were in the COG category of unknown function.

## 3. Discussion

Promising therapeutical features of nematode parasites are a consequence of the long-term coevolution of the host–parasite relationship. This relationship has resulted in the development of a unique type of immune modulation and tolerance induced by the parasite, which is beneficial for the host, including humans. Immune modulatory activity of nematodes mostly results in T helper cell type 2 (Th2) and T regulatory cell (Treg) induction and T helper cells type 1 (Th1) inhibition [15]. However, many different mechanisms of immune modulation by parasites have been described so far [16]. Nevertheless, the exact effect of parasitic nematodes presence on the immune system of the host depends on molecules produced by them. To understand the phenomenon of helminth therapy, we evaluated a set of these factors produced by *H. polygyrus* that developed in mice with DSS-induced colitis.

The course of the inflammation of the colon in mice infected with *H. polygyrus* was analogous to that observed in our previous experiments and to parasitic infection-induced protection against DSS-induced colitis [17]. Here, we observed that *H. polygyrus* L4 stage that developed in the colitic milieu produced a higher number of proteins than nematodes isolated from “healthy” mice. This phenomenon concerned both males and females. Dioeciousness is observed in most nematode species. There are significant differences in ES composition between male- and female-derived products including *H. polygyrus* species [12,14,18,19,20]. However, the protein ratio of HpC male to Hp male was higher than the ratio of HpC female to Hp female proteins, respectively. One reason for such a big difference in the number of identified proteins between two evaluated groups of ES produced by male could be their size. In control conditions, when nematodes develop in healthy mice, male *H. polygyrus* are significantly smaller than female. In our previous studies, we showed that male nematodes that developed in the colitic microenvironment are larger and reach sizes comparable to those of female nematodes [11].

Nematodes developed during colitis produced proteins that have not been observed in other studied groups. In the ES of HpC females, we identified several proteins including annexin, lysozyme-2, apyrase, and galectin, among others. In the secretome of HpC males, we found unique proteins classified as venom allergen/Ancylostoma secreted protein-like, transthyretin-like family proteins, and galectins. These molecules could be responsible for the therapeutic effect of the nematode in DSS-induced colitis.

Among the unique proteins produced by female *H. polygyrus* L4 stage, annexin was found to be highly abundant. Annexins play a significant role in various biological processes, including the immune response [21]. Parasitic annexins are involved in the host–parasite relationship and are immunomodulators [22]. *Taenia solium*, an etiological agent of taeniasis, produces annexin B1, which induces calcium-dependent apoptosis in human eosinophils [23]. *Schistosoma bovis* annexins exhibit fibrinolytic and anticoagulant properties [24]. In addition, annexins produced by helminths are considered promising candidates for vaccines or drugs against parasitic invasion development [22]. The function of annexins produced by nematodes infecting mammals has not been described yet. However, it is likely that annexins play a part in important immunomodulatory processes, as they are often identified in parasitic nematode genomes or secretomes. Annexin-coding genes are present in *Brugia malayi* [25]. Annexins have also been identified in the secretomes of *Angiostrongylus cantonensis* and *Ascaris suum* [26,27].

Lysozymes are produced by various nematodes. The unique lysozyme production observed in the HpC female group suggests that female nematodes can influence the intestinal microbiota of the host. Nematodes produce molecules with antibacterial activity [28,29]; hence, parasitic invasion can modify the microbiota composition. This phenomenon has also been confirmed in mice infected with *H. polygyrus* and hookworm used in clinical trials of helminth therapy [30,31,32]. Restoring the normal bacterial microflora is one of the methods of treating IBD [33]. Therefore, lysozyme production by female nematodes could be a contributing factor to the therapeutic effect of helminth therapy.

A unique apyrase has also been identified in the ES of females that developed in the colitis microenvironment. Apyrases are enzymes produced by various parasitic worm species [34,35,36]. A potential function of the apyrases produced by nematodes is the hydrolysis of extracellular ATP. Extracellular ATP is an example of an alarmin that can induce an antiparasitic T helper type 2 immune response and cellular production of IL-33 [34]. Mice immunized with an apyrase cocktail produced by *H. polygyrus* showed a partial protective effect against a second invasion, which points to their immunogenicity [34]. In our study of the immunogenicity of *H. polygyrus* L4 stage that developed during colitis, nematodes in mice with colitis, especially females, induced higher levels of specific antibodies; however, we did not identify any apyrase in the immunoproteome of HpC females [12].

Unique galectins have been identified in the ES of both males and females that developed during colitis. Galectins bind β-galactosides and are involved in a variety of processes, including immune defense [37]. Various nematode species produce galectins [38], and these galectins produced by parasites may have an immunomodulatory function. *Angiostrongylus cantonensis* gal-1 induces apoptosis of macrophages through the apoptotic signaling pathway activation and is dependent on annexin A2 [39]. In our previous studies, we found that galectin from *Teladorsagia circumcincta* modified mast cell activity [40]. It is an immunomodulatory mechanism used by nematodes to enhance their establishment and survival [40]. Hence, the presence of galectins in the secretome of nematodes may contribute to the therapeutic effect, especially considering the role of mast cells in contributing to IBD pathogenesis [41].

Among the unique molecules observed in the ES of the HpC male group, there were proteins belonging to venom allergen-like proteins (VALs). VALs are members of sperm coating-like protein/Tpx-1/antigen 5/pathogenesis-related-1/Sc7 (SCP/TAPS) protein family. VALs shows homology to activation-associated proteins (ASPs) that have been observed in *Ancylostoma caninium* and other strongylid species [42]. VALs appear to be produced at times of the parasite life cycle when there is the most contact between parasite and host, whether through transmission, tissue migration, or feeding [43]. Some VALs are directly involved in immunomodulation. Na-ASP-2 from hookworm, *N. americanus*, binds to human B cells and affects the antigen receptor pathway [44]. The same protein possesses the ability to recruit neutrophiles and monocytes [45]. SmVAL9, produced by trematode species *Schistosoma mansoni*, affects the expression of extracellular matrix, remodeling matrix metalloproteinase (MMP) and tissue inhibitors of metalloproteinase (TIMP) in macrophages [46].

Transthyretin-like family proteins are members of a highly conserved proteins group found only in the phylum Nematoda. These proteins show sequence similarity to the transthyretins and transthyretin-related proteins [47]. Transthyretin-like family proteins are excreted/secreted by various parasitic nematode species [27,48,49], but their participation in immunomodulation has not been confirmed yet.

The proteins identified in this work are consistent with previous studies aimed at identifying candidates from *Ancylostoma caninum* ES as inflammatory bowel disease therapeutics. The analysis was based on statistical filtering and ranking and resulted in 20 protein indications, including annexins, transthyretins, SCP/TAPS, and lysozyme [50]. It indicates a general result of this type of investigation, despite variation among parasite species and research methods used.

Microbiota dysbiosis is one of the factors that contribute to the development of inflammatory bowel diseases [51]. The proteins identified in this study have the potential to directly or indirectly impact intestinal microbiota via regulating immune cells. Parasitic nematodes, on the other hand, are likely to change the host’s immune response precisely through their influence on the microflora. The link between the microbiome and the macrobiome has been observed, and bacteria and parasites are known to influence each other [52].

In conclusion, we confirmed that modification of the intestinal milieu significantly influences nematode ES proteome composition. This finding is extremely important in understanding helminth therapy mechanisms. Moreover, the study identified new, unique molecules produced by the parasite introduced into inflammatory conditions characteristic of inflammatory bowel diseases. It is important to test individual proteins indicated in this work or to construct a recombinant protein based on these findings. They may apply to inflammatory bowel disease, but also to other inflammatory disorders.

## 4. Materials and Methods

### 4.1. Parasite Preparation for In Vitro Culture

All experimental procedures were performed in accordance with the Polish Law on Animal Experimentation and Directive 2010/63/UE. Eight-week-old pathogen-free BALB/c males were allowed to adapt to the laboratory environment one week before the experiment began. Acute severe colitis was induced by the administration of 3% dextran sulphate sodium (DSS) (TdB Consultancy AB, Uppsala, Sweden), a 35–50 kDa sulphated polymer, in drinking water. The DDS was administered two days before oral infection with 300 L3 of *H. polygyrus*, and administration of DSS was continued until the end of the experiment. Successful induction of colitis was confirmed as previously described [13]. Mice were divided into two experimental groups: the first group of mice was treated with DSS and the second group untreated. *H. polygyrus* (HpC) was collected from the DSS-treated mice, and *H. polygyrus* (Hp) was collected from the untreated mice. The mice were euthanized six days after infection, when the parasite was in its fourth larval stage. The larvae were collected and the sex of the L4 stage was determined as described before [53]. Two separate experiments were carried out on ES generated from nematode cultures collected from independently infected mice.

### 4.2. Excretory–Secretory Molecules Obtainment

Nematodes were cultured as previously described [50]. Briefly, *H. polygyrus* L4 stage forms were cultured at a density of 600 larvae per ml in RPMI 1640 medium (Biowest, Nuaillé, France) supplemented with L-glutamine 2 mM (Biowest, Nuaillé, France), penicillin (100 U/mL), and streptomycin (100 μg/mL), without fetal bovine serum (FBS), at 37 °C. The first 24 h’s collection of ES was discarded; the medium was supplied and harvested for the next 72 h. The supernatant was collected, centrifuged, and concentrated; then, it was sterilized through 0.2 μm filters. The final protein concentration of samples was measured using RC DC™ Protein Assay (Bio-Rad, Hercules, CA, USA) and stored at −80 °C until analysis.

### 4.3. Sample Preparation

The mass spectrometry analysis was performed at the Mass Spectrometry Laboratory IBB PAS as described previously [14]. Two independent analyses of the ES samples were conducted.

### 4.4. Analysis of Mass Spectrometry Results and Protein Identification

The acquired MS/MS data were preprocessed with Mascot Distiller software (v. 2.6 or 2.7, MatrixScience, London, UK), and a search was performed with the Mascot Search Engine (MatrixScience, London, UK, Mascot Server 2.5) against the Nematoda proteins (1,283,514 sequences) deposited in NCBInr database (20190409, 198,058,131 sequences; 72,054,367,693 residues). To reduce mass errors, the peptide and fragment mass tolerance settings were established separately for individual LC-MS/MS runs after a measured mass recalibration. Methylothiolation (C) was set as a fixed modification and oxidation (M) as a variable modification. Peptide mass tolerance and fragment mass tolerance were set at ±5 ppm and ±0.01 Da, respectively. The rest of the search parameters were as follows: enzyme: trypsin; missed cleavages: one; and instrument: HCD. The Decoy option was activated for further target/decoy-based FDR control, and the peptide score threshold was adjusted to maintain the FDR at <1%. The significance threshold was set at *p* < 0.01. The results were filtered using the Mascot Percolator. Proteins with score values of at least 70 were analyzed. One representative of two analyses of the ES is presented.

### 4.5. Bioinformatic Analysis

Protein functional annotation was performed using OmicsBox v. 2.4.4 software (Biobam, Valencia, Spain). The initial Blast search was conducted against the NCBI non-redundant (nr) database, with default parameters. Gene Mapping and Gene Ontology (GO) annotations were made with default parameters. InterPro annotations were made with the analysis of signal peptides in SignalP, while the remaining settings were set to default. GO annotation results were merged with the InterPro database scanning results and EggNOG v5.0 orthology and functional annotation data. A Venn diagram was prepared using InteractiVenn [54]. A heatmap with shared proteins emPAI values was prepared using GraphPad Prism 8.

## Figures and Tables

**Table 1:** Twenty most abundant unique proteins identified in excretory–secretory molecules of *H. polygyrus* L4 stage males that developed during colitis (HpC males).

NCBI ID	Description	MASCOT Score	emPAI
VDL71588.1	Tubulin beta chain	1257	1.5
VDP22131.1	EF-hand domain-containing protein	141	1.42
VDP34397.1	HIT domain-containing protein	186	1.24
VDO18942.1	Cystatin domain-containing protein	351	1.1
KJH43834.1	EF hand	202	0.95
VDO89063.1	Aldo_ket_red domain-containing protein	395	0.88
AEP82923.1	Venom allergen/ancylostoma secreted protein-like 7 isoform 2	376	0.87
AAQ01172.1	Tubulin beta chain	200	0.83
VDO28252.1	HTH cro/C1-type domain-containing protein	96	0.82
VDO76677.1	Ground-like domain-containing protein	110	0.75
VDP14233.1	Transthyretin-like family protein	146	0.73
VDP54111.1	Charged multivesicular body protein 5	140	0.73
VDO99034.1	Uncharacterized protein	202	0.72
VDP30992.1	FABP domain-containing protein	123	0.72
VDP61579.1	HYPK_UBA domain-containing protein	104	0.61
CAD11862.1	Troponin	78	0.58
VDO61101.1	SGNH domain-containing protein	207	0.55
VDP46572.1	Neur_chan_memb domain-containing protein	77	0.53
VDO22880.1	Uncharacterized protein	184	0.51
PAV68437.1	Actin	337	0.49

**Table 2:** Twenty most abundant and unique proteins identified in excretory–secretory molecules of *H. polygyrus* L4 stage females that developed during colitis (HpC females).

NCBI ID	Description	MASCOT Score	emPAI
VDO94699.1	Aldo_ket_red domain-containing protein	129	1.09
VDP21402.1	SCP domain-containing protein	241	0.87
VDP57907.1	Annexin	138	0.83
VDM52930.1	A2M_N_2 domain-containing protein	138	0.83
VDP37313.1	Peptidylprolyl isomerase	75	0.72
VDO20172.1	VOC domain-containing protein	113	0.61
CCC54340.1	Lysozyme-2	146	0.52
EYC45310.1	Tubulin beta chain	483	0.49
VDP33044.1	Spherulation-specific family 4	121	0.4
VDP19498.1	Apyrase	104	0.39
PIO69298.1	Fructose-bisphosphate aldolase	358	0.38
VDO92704.1	ADF-H domain-containing protein	144	0.38
2OS5_A	Chain A. AceMIF	82	0.37
VDP05536.1	Aldo_ket_red domain-containing protein	82	0.36
VDP08224.1	Sushi domain-containing protein	134	0.34
VDP05504.1	Galectin	86	0.32
VDL70545.1	Transthyretin-like family protein	83	0.3
VDO70571.1	EB domain-containing protein	70	0.29
VDP10806.1	Peptidase A1 domain-containing protein	107	0.26
VDP12486.1	Sulfurtransferase	84	0.25

## Data Availability

Data supporting the conclusions of this article are included within the article. The mass spectrometry proteomics data have been deposited to the ProteomeXchange Consortium via the PRIDE [55] partner repository with the dataset identifier PXD041516 and DOI 10.6019/PXD041516.

## Supplementary Materials

The following supporting information can be downloaded at: https://www.mdpi.com/article/10.3390/ijms241814127/s1.
